# A case of Shwachman–Diamond syndrome

**DOI:** 10.1186/1687-9856-2013-S1-P43

**Published:** 2013-10-03

**Authors:** Ji Hoon Kim, Won Kyoung Cho, Tai-sung Kim, Yun-Jung Choi, Byung-Kyu Suh

**Affiliations:** 1 Department of Pediatrics, College of Medicine, The Catholic University of Korea

## 

Shwachman–Diamond syndrome (SDS) is an autosomal recessive disorder (OMIM 260400), characterized by exocrine pancreatic insufficiency, skeletal abnormalities and bone marrow (BM) dysfunction, with a risk, as high as 30%, to develop myelodysplastic syndrome and/or acute myeloid leukaemia (MDS/AML). The SBDS gene (OMIM 607744) is localized on chromosome 7 at the band q11 and mutations of this gene are found in 90% of patients.

Direct sequencing of whole exon 2 and flanking intronic regions of the *SBDS* gene was performed on an ABI Prism 3100 Genetic Analyzer (Applied Biosystems, USA) using a BigDye Terminator v3.1 Cycle Sequencing Kit (Applied Biosystems). The forward (F) and reverse (R) primers for amplifying exon 2 are AAATGGTAAGGCAAATACGG (2Fa), AGACCTCGATGAAGTTCT-GC (2Fb) and ACCAAGTTCTTTATTATTAGAAGTGAC (2R).

We described a 14 years old boy who presented with skeletal system abnormalities (Legg-Calve-Perthes syndrome, congenital coxa vara and genu valgum deformity), short stature, chronic dydpepsia, neutropenia and thrombocytopenia. Abdominal CT of patient showed congenital lipomatosis of pancreas and spine bone density shows osteoporosis. Direct sequencing for whole exons including intron-exon boundaries of patient showed two heterozygous mutations, c. [183_184TA>CT] + [258+2T>C].

We treated the patient with pancreatin (Now Foods Pancreatin 1T=Lipase 9,000/Amylase 50,000/Protease 50,000 USP units) and Dicamax (1T=Ca. carbonate 1,250 mg and cholecalciferol 1,000 IU). We has observed for hematologic abnormality and prepared for bone marrow transplantation.

We report a child with diverse clinical manifestations of SDS including short stature, chronic dydpepsia, skeletal system abnormalities, and neutropenia; the clinical diagnosis was confirmed by genetic analysis for the second time in Korea.

